# ARV1 is a component of the enzyme initiating glycosylphosphatidylinositol biosynthesis

**DOI:** 10.1016/j.jbc.2025.110236

**Published:** 2025-05-14

**Authors:** TianTian Lu, Saori Umeshita, Kae Imanishi, Yicheng Wang, Yi-Shi Liu, Masamichi Nagae, Yuya Senoo, Kazutaka Ikeda, Morihisa Fujita, Taroh Kinoshita, Yoshiko Murakami

**Affiliations:** 1 Laboratory of Immunoglycobiology, Research Institute for Microbial Diseases, Osaka University, Osaka, Japan; 2 Immunology Frontier Research Center, Osaka University, Osaka, Japan; 3 Key Laboratory of Carbohydrate Chemistry and Biotechnology, School of Biotechnology, Jiangnan University, Wuxi, Jiangsu, China; 4 Laboratory of Biomolecule Analysis, Department of Applied Genomics, Kazusa DNA Research Institute, Chiba, Japan; 5 Institute for Glyco-core Research (iGCORE), Gifu University, Gifu, Japan; 6 Center for Infectious Disease Education and Research, Osaka University, Osaka, Japan

**Keywords:** AlphaFold, GPI N-acetylglucosaminyltransferase complex, inherited GPI deficiency, lipidomics, phosphatidylinositol

## Abstract

Glycosylphosphatidylinositol (GPI) serves as a membrane anchor of numerous cell surface proteins. It is synthesized in the endoplasmic reticulum from phosphatidylinositol (PI) by stepwise reactions and transferred to the C terminus of the protein. Defects in genes involved in GPI biosynthesis affect the expression of GPI-anchored proteins or their structure, causing the neurological disorder, inherited GPI deficiency. Individuals with ARV1 deficiency have symptoms resembling inherited GPI deficiency, but how ARV1 regulates GPI biosynthesis is poorly understood. Here, we show that ARV1 acts as a component of the enzyme initiating GPI biosynthesis, GPI N-acetylglucosaminyltransferase (GPI–GnT) complex, which forms a ring structure as predicted by AlphaFold3. ARV1 associates with PIGQ, a GPI–GnT component, and ARV1 mutants defective in this association lose their ability to enhance GPI–GnT activity, showing that association with PIGQ is critical for ARV1’s function. ARV1-containing GPI–GnT used PI more efficiently than ARV1-less GPI–GnT in an *in vitro* enzyme assay. Collectively, our results suggest that ARV1 facilitates efficient recruitment of PI to GPI–GnT, thereby playing a critical role in the regulation of GPI-anchored protein expression.

Glycosylphosphatidylinositol (GPI) is a glycolipid covalently attached to the C terminus of specific proteins as a membrane anchor. GPI anchoring of proteins is ubiquitous in eukaryotes. In humans, more than 150 proteins with various functions, such as enzymes, receptors, complement regulators, and adhesion molecules, have been identified as GPI-anchored proteins (GPI-APs) (1, 2). Free-form GPIs not linked to proteins have also been found in mammalian cells and tissues, indicating that GPI might be involved in more biological processes besides acting as a protein membrane anchor (3, 4).

The core structure of GPI is well conserved among the eukaryotes (1). The biosynthetic pathway of GPI is a stepwise process involving more than 10 steps. It is initiated on the cytoplasmic side of the endoplasmic reticulum (ER) by GPI N-acetylglucosaminyltransferase (GPI–GnT), which catalyzes the transfer of GlcNAc from UDP-GlcNAc to phosphatidylinositol (PI) to generate GlcNAc–PI, followed by de-N-acetylation by PIGL to generate glucosamine (GlcN)-PI (5, 6). There are seven known subunits in the human GPI–GnT complex: PIGA, PIGC, PIGH, PIGP, PIGQ, PIGY, and DPM2 (7). PIGA belongs to the glycosyltransferase (GT) family 4, acting as a catalytic subunit of the enzyme complex. Other subunits such as PIGQ and PIGC may help to stabilize the complex and regulate its enzymatic activity (8, 9), but how these subunits function remains unclear.

At least 30 genes are involved in the biosynthesis of GPI-AP. Germline mutations in these genes cause inherited GPI deficiency (IGD), major symptoms of which are neurological disorders such as seizures, cerebral and/or cerebellar atrophy, and hearing and/or visual impairment (10, 11). These symptoms are caused by the decreased expression or abnormal anchor structures of GPI-APs due to the partial loss of function of genes involved in GPI biosynthesis.

ARV1 (ARE2 required for viability 1) (12, 13) is a multitransmembrane protein in the ER of various organisms (13, 14, 15). It was first identified in the yeast *Saccharomyces cerevisiae* as a sterol homeostasis mediator, being required for viability in the absence of acyl CoA:cholesterol acyltransferase (encoded by *ARE1* and *ARE2* genes) (13). Yeast lacking ARV1 has increased levels of free sterol and sterol ester, increased ER sterol, and decreased plasma membrane sterol, showing abnormal sterol distribution (13). ARV1-lacking yeast is also defective in sphingolipid metabolism with the accumulation of ceramide in the ER (16). The biosynthesis of phospholipids (16) and GPI (17, 18) is also affected by ARV1 deficiency. These abnormalities of lipid metabolism in the ER of ARV1-defective yeast are associated with protein-misfolding–independent induction of the unfolded protein response (19). Furthermore, yeast lacking ARV1 is highly sensitive to toxicity of free unsaturated fatty acids (20). The mechanistic basis for these various lipid-related phenotypes is not well understood, although it was recently reported that *Candida albicans* ARV1 physically associates with and stabilizes ERG11, the rate-limiting enzyme in sterol biosynthesis (21).

Mammalian ARV1 can rescue lipid-related defects in yeast lacking ARV1, indicating that mammalian ARV1 plays a similar role in lipid homeostasis (13). Indeed, ARV1 knockdown in cultured cells caused the accumulation of cholesterol in the ER and decreased plasma membrane cholesterol (22), decreased triglyceride synthesis, increased fatty acid sensitivity (20), and activated the unfolded protein response (19). In addition, the overexpression of ARV1 in HEK293 cells caused increases in triglycerides and the number of lipid droplets (20). Meanwhile, mice with *Arv1* gene knockout had a lean phenotype, shrunken adipose tissues, low blood cholesterol and triglyceride, and higher consumption of glucose, suggesting that ARV1 is important for fatty acid homeostasis and the regulation of energy expenditure in mice (23, 24).

In 2015 and 2016, three children with homozygous *ARV1* mutation in a consanguineous family were reported to have autosomal recessive epileptic encephalopathy (25, 26). Five years later, 14 more individuals harboring biallelic variants in *ARV1* were reported (27, 28), upon which this disease was named early infantile epileptic encephalopathy 38 (EIEE38, MIM#617020). EIEE38 patients share many symptoms with IGD, including neurodevelopmental delay, hypotonia, intractable infantile-onset seizure disorder, and early death. Decreased expression of GPI-APs on fibroblasts and neutrophils was also reported (27, 28).

A report on computational model structures of protein complexes in yeast suggested that GPI1 (homolog of human PIGQ) associates with yeast ARV1 (29). Elsewhere, Ji *et al.* reported their proteomic characterization of *Trypanosoma brucei* GPI–GnT, including the finding that trypanosomal Arv1 and UbCE are potential subunits of this complex (30). We also reported the association of human ARV1 and PIGQ *via* an experiment using their fusion proteins with split fragments of the fluorescent protein mCitrine (31). Here, we show that human ARV1 enhances the enzymatic activity of the GPI–GnT complex by associating with PIGQ. Loss of ARV1 causes reduced GlcNAc–PI production.

## Results

### ARV1 is involved in the first step of GPI biosynthesis

**Figure 1:**
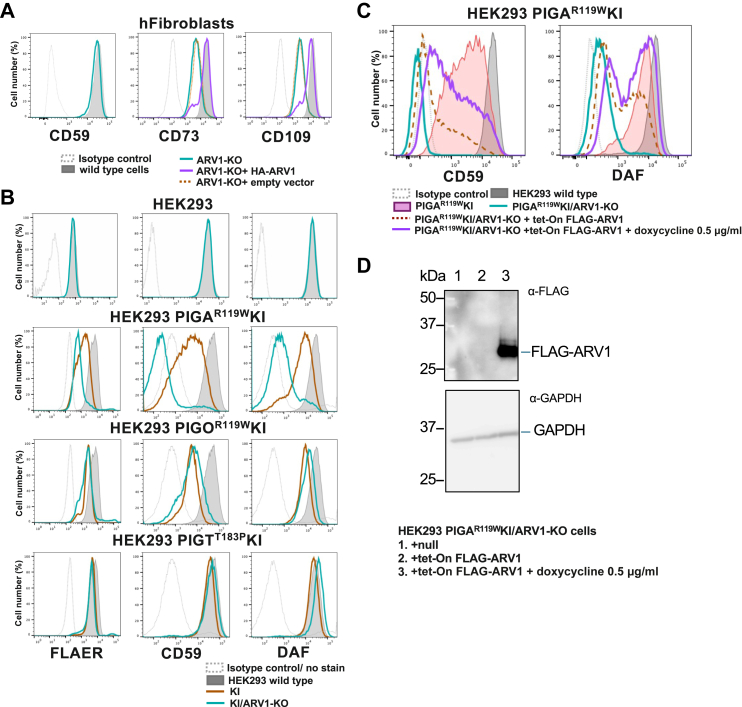
**ARV1-deficient human cells exhibit decreased expression of GPI-APs**. *A*, flow cytometry (FACS) analysis of human fibroblasts. WT (*gray*), ARV1-KO (*blue*), and ARV1-rescued (*purple*) or vector-transfected (*orange* dotted) ARV1-KO cells were stained for GPI-APs. Mean fluorescence intensity (MFI) of CD59, CD73, and CD109 expressed on ARV1-KO fibroblasts decreased to 80%, 25%, and 18% of WT levels, respectively. *Gray* dotted lines: isotype control staining. *B*, *top* panel. FACS analysis of HEK293 cells. FLAER staining and CD59 and DAF levels on ARV1-KO HEK293 cells (*blue*) were not decreased from those on WT HEK293 cells (*gray*). *B*, three *bottom* panels. FACS analysis of PIGA^R119W^KI, PIGO^R119W^KI, and PIGT^T183P^KI HEK293 cell lines with further KO of ARV1. Expression of GPI-APs was decreased only in PIGA^R119W^KI cells by further KO of ARV1. *C*, FACS analysis of PIGA^R119W^KI/ARV1-KO HEK293 cells, stably expressing FLAG-ARV1 by the tet-On system, showed partial rescue of CD59 and DAF expression. *D*, Western blotting analysis of FLAG-ARV1 (34 kDa). GAPDH: a loading control. Each FACS analysis was performed at least two times.

To establish a causal relationship between ARV1 defect and GPI-AP deficiency found in patients with EIEE38 (27) and to clarify its mechanistic basis, knockout of ARV1 was performed in human fibroblasts and HEK293 cells, and cell surface levels of GPI-APs were determined. ARV1-KO in fibroblasts did not severely affect the CD59 level but caused clear decreases in CD73 and CD109 levels, which were restored by the stable expression of ARV1 cDNA (Fig. 1*A*). These results suggest that ARV1 is not essential for GPI-AP biosynthesis but is critical for maintaining normal GPI biosynthesis levels in human cells. Similar to CD59 on fibroblasts, ARV1-KO in HEK293 cells did not affect CD59 and DAF levels or the staining intensity of fluorescently labeled aerolysin (FLAER), which binds to various GPI-APs (Fig. 1*B*), suggesting that a decreased level of GPI anchors under ARV1-deficient conditions is sufficient for normal level expression of various GPI-APs in HEK293 cells.

Next, we generated a system to determine the step in the GPI-AP biosynthetic pathway in which ARV1 functions. To determine the effect of ARV1-KO, we generated HEK293 cell lines bearing hypomorphic mutations of genes involved in the GPI biosynthetic pathway using *CRISPR*/*Cas9*-mediated knock-in *(KI*) because the negative influences of related genes might amplify their effects. We employed three mutant HEK293 cells, PIGA^R119W^KI (32), PIGO^R119W^KI (33), and PIGT^T183P^KI (34); PIGA, PIGO, and PIGT function in the first, eighth, and tenth steps of the GPI biosynthesis pathway, respectively. At least one of the three alleles of each PIG gene in these mutant HEK293 cells was knocked in with the indicated hypomorphic variants derived from IGD patients, whereas other alleles were knocked out during the KI procedure. The levels of CD59, DAF, and FLAER staining were reduced due to the hypomorphic nature of the knocked-in variants (Fig. 1*B*). In these gene-manipulated cells, ARV1 was knocked out. The levels of FLAER staining, CD59, and DAF on PIGA^R119W^KI cells were greatly reduced or nearly completely lost by the KO of ARV1 (Fig. 1*B*). Meanwhile, the CD59 and DAF levels were restored by the stable expression of ARV1 cDNA (Fig. 1, *C* and *D*). In contrast, the KO of ARV1 in PIGO^R119W^KI and PIGT^T183P^KI cells did not cause further reduction in FLAER staining or CD59 and DAF levels (Fig. 1*B*). These results suggested that ARV1 regulates the first step of the GPI biosynthetic pathway, which is consistent with the physical association of ARV1 and GPI–GnT complex in trypanosomes (30).

### ARV1 associates with GPI–GnT complex *via* PIGQ

**Figure 2:**
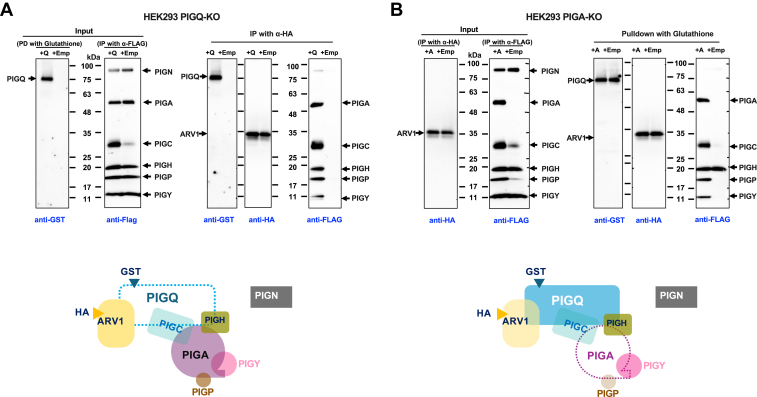
**ARV1 directly associates only with PIGQ in GPI–GnT complex**. *A*, *Upper*: Co-IP assay (IP with HA) using PIGQ-KO HEK293 cells. PIGQ-KO cells with (+) or without (−) rescue of GST-PIGQ were transiently transfected with HA-ARV1 and other FLAG-tagged subunits of GPI-GnT and PIGN, as a negative control. *Left* two panels (Input): Expression of transfected proteins. *Right* three panels (IP: HA beads): ARV1-bound GPI–GnT complex only through GST-PIGQ. *Lower*: Schematic of predicted GPI–GnT complex in PIGQ-KO cells. *B*, *Upper*: Co-pull-down assay (pull-down with GST) using PIGA-KO HEK293 cells. PIGA-KO cells with (+) or without (−) rescue of FLAG-PIGA were transiently transfected with HA-ARV1, GST-PIGQ, and other FLAG-tagged subunits and PIGN. *Left* two panels (Input): Expression of transfected proteins. *Right* three panels (pull-down: glutathione-Sepharose): PIGQ bound to ARV1 and PIGH in the absence of PIGA. *Lower*: Schematic of predicted GPI–GnT complex in PIGA-KO cells. For (*A* and *B*), experiments were performed at least three times.

Human GPI–GnT consists of seven subunits, PIGA, PIGC, PIGH, PIGQ, PIGP, PIGY, and DPM2. To investigate how ARV1 associates with the GPI–GnT complex, we focused on its association with PIGQ because we previously found an association between their fusion proteins using split mCitrine (31). To test whether ARV1 associates with GPI–GnT *via* PIGQ, co-immunoprecipitation analysis was performed using PIGQ-KO (Fig. 2*A*) or PIGA-KO (Fig. 2*B*) HEK293 cells. In PIGQ-KO cells transfected with HA-ARV1 together with FLAG-tagged GPI–GnT components, all components except PIGC were expressed stably (Fig. 2*A*, right lane in right “Input” panel), but no component was coprecipitated with HA-ARV1 (right lane in right “IP” panel). However, when PIGQ was expressed using glutathione S-transferase (GST)-tagged PIGQ (GST-PIGQ), PIGC expression was restored (Fig. 2*A*, left lane in right “Input” panel) and all of the FLAG-tagged components, except negative control PIGN, were coprecipitated with HA-ARV1 (left lane in right “IP” panel), suggesting that ARV1 directly associates only with PIGQ (bottom in Fig. 2*A*). In PIGA-KO cells transfected with GST-PIGQ together with HA-ARV1 and other FLAG-tagged GPI–GnT components, the expression of PIGC and PIGP was inefficient (Fig. 2*B*, right lane in Input panel) and only HA-ARV1 and FLAG-PIGH were coprecipitated with GST-PIGQ (right lanes in right “IP” panels). However, when PIGA was restored using FLAG-PIGA, all of the components were expressed efficiently and were coprecipitated with GST-PIGQ (left lanes in right “Input” panel and left lanes in “Pulldown” panels), suggesting that PIGQ directly associates with ARV1 and PIGH despite the GPI–GnT complex being disrupted (bottom in Fig. 2*B*). At this point, we concluded that ARV1 is the eighth component of the human GPI–GnT complex and that it associates with this complex *via* PIGQ.

### ARV1 contributes to GPI biosynthesis by associating with PIGQ on the cytoplasmic side of the ER

**Figure 3:**
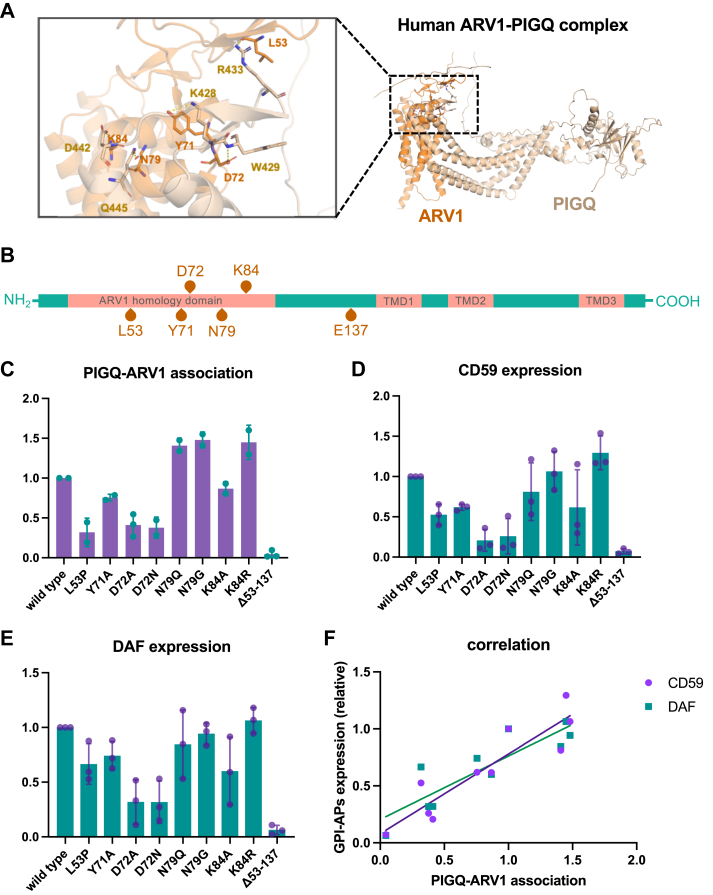
**ARV1 mutants showing reduced association with PIGQ have decreased GPI–GnT enzyme activity.***A*, a model of human ARV1 and PIGQ complex predicted by AlphaFold2. Candidate amino acids for protein association are shown with their structures. ARV1, *orange*; PIGQ, *khaki*; oxygen atom, *red*; nitrogen atom, *blue*; hydrogen bond, *yellow* dotted line. *B*, domain structure of ARV1 protein. Candidate amino acids for ARV1-PIGQ association are marked. *C*, the relative association between HA-ARV1 mutants and FLAG-PIGQ calculated from the results of co-IP assay (pull-down with FLAG) (Fig. S2). Band intensities in Western blotting were quantified using ImageJ (version 2.3.0/1.53q). The relative amounts of HA-ARV1 mutants were normalized by FLAG-PIGQ amounts. *D* and *E*, relative expression level of GPI-APs in ARV1-rescued cells. HA-ARV1 mutants were transiently expressed in PIGA^R119W^KI/ARV1-KO HEK293 cells, and the expression levels of CD59 (*D*) and DAF (*E*) were analyzed by FACS (Fig. S2). MFI was quantified using FlowJo 10.9.0. *F*, correlation of GPI-AP expression levels in ARV1 mutant rescued cells and relative association between ARV1 mutants and PIGQ. Pearson r = 0.91 for association *versus* CD59 expression (*p* value, 0.0002), Pearson r = 0.87 for association *versus* DAF expression (*p* value, 0.001). For (*C*–*E*), experiments were performed at least three times, analyzed by one sample *t* test and the error bars are SD. TMD, transmembrane domain.

To investigate the interaction between PIGQ and ARV1 in more detail, a model of the interaction of human PIGQ and ARV1 was created using AlphaFold2. We found that the cytosolic parts of PIGQ (khaki) and the ARV1 homology domain (AHD) in ARV1 (orange) possess several potential binding sites (Figs. 3*A* and S1*C*). Among the candidate binding sites, D72 and K84 of human ARV1 are conserved with D48 and K53 in yeast ARV1, respectively (Fig. S1, *A*–*C*). Co-immunoprecipitation experiments were performed to detect the association between FLAG-PIGQ and HA-ARV1 mutants, aiming to confirm the validity of the prediction. FLAG-PIGQ was pulled down by anti-FLAG beads, and the PIGQ–ARV1 complex was detected by anti-HA antibody on a Western blot (Fig. S2*A*). A region containing all potential interacting amino acids was first deleted from ARV1, generating ARV1▵53–137 (Fig. 3*B*). The ARV1▵53–137 mutant was efficiently expressed but lost the ability to associate with PIGQ (Figs. 3*C* and S2*A*). Next, single amino acid substitutions were introduced to individual predicted binding sites. ARV1^L53P^, ARV1^Y71A^, ARV1^D72N/A^, and ARV1^K84A^ lost some of their ability to interact with PIGQ, while ARV1^N79G/Q^ and ARV1^K84R^ maintained their binding ability (Figs. 3*C* and S2*A*). The predicted model contains a hydrogen bond between the side-chain carboxyl of ARV1^D72^ and nitrogen atoms in the PIGQ backbone, while this bond was lost when the acidic carboxyl was changed to amidogen in ARV1^D72N^. Similarly, the amino group of ARV1^K84^ can form a salt bridge with the carboxyl group of PIGQ^D442^, and the affinity was reduced when lysine was mutated to alanine, but the bond still existed when lysine was changed to arginine (Figs. 3*C* and S2*A*). These results suggest that the prediction was valid.

To explore whether the mutations in ARV1 proteins influence the overall activity of GPI–GnT, these ARV1 mutants were transiently expressed in HEK293 PIGA^R119W^KI/ARV1-KO double-mutant cells (Fig. S2*B*). These ARV1 mutants were able to restore the expression of GPI-APs to varying degrees (Figs. 3, *D* and *E*, and S2*B*). Quantitative findings show that the activity of GPI–GnT with ARV1 mutant has a good correlation with the ARV1-PIGQ association levels, indicating that the interaction of ARV1 with GPI–GnT is important for the enzymatic activity of this complex (Fig. 3*F*).

### ARV1 enhances enzyme activity of GPI–GnT

**Figure 4:**
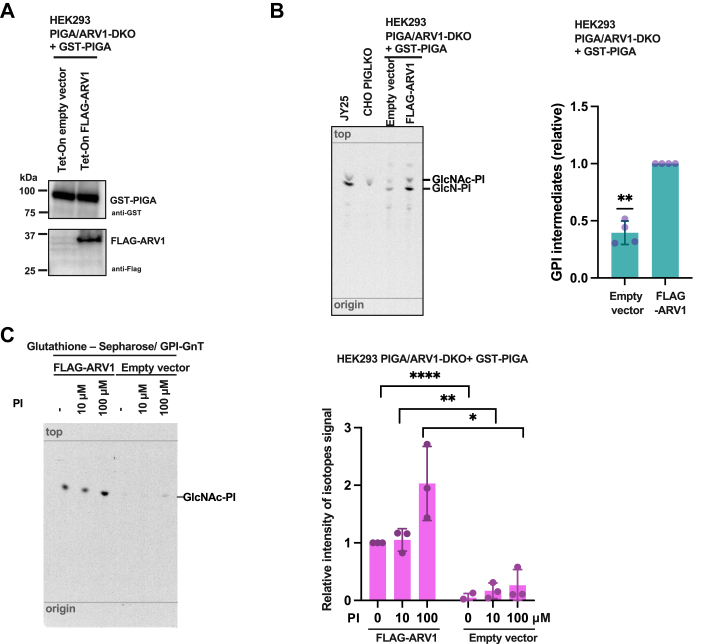
**ARV1-containing GPI-GnT showed stronger activity than ARV1-less-GPI-GnT in *in vitro* enzyme assays.***A*, Western blotting of PIGA/ARV1-DKO HEK293 cells stably expressing GST-PIGA (*top panel*) with or without tet-On system-controlled expression of FLAG-ARV1 (*bottom panel*). *B*, *Left*: High-performance thin-layer chromatography (HPTLC) of butanol-extracted radiolabeled products from enzyme assay using cell lysates. Products from JY25 cells (human WT B-lymphoblasts) and PIGL-KO Chinese hamster ovary (CHO) cells were used as standards for GlcNAc-PI and GlcN-PI (50). Developing solvent, chloroform/methanol/1 M NH_4_OH = 10:10:3 (v:v:v) (9). *Right*: Amounts of radioactive products (GlcNAc-PI + GlcN-PI intensities, normalized by phosphatidylcholine levels detected by molybdenum blue reagent). *p* value, 0.0045 by one sample *t* test. *C*, *Left*: HPTLC of products from enzyme assay using purified ARV1-containing (*left half*) and ARV1-less (*right half*) GPI–GnT complexes with PI at three different concentrations. *Right*: Quantification of GlcNAc-PI intensities. *p* value, 0.00002 with 0 μM PI; 0.003 for 10 μM PI; 0.01 for 100 μM PI by Student's *t* test. For (*B* and *C*), assays were performed at least three times. The error bars are SD.

To compare cellular GPI–GnT enzyme activities in the presence and absence of ARV1, an *in vitro* enzyme assay was applied to the cell lysates. The enzyme sources were PIGA/ARV1-double-knockout (DKO) HEK293 cells, into which GST-PIGA and FLAG-ARV1 (or empty vector) were permanently transfected (Fig. 4*A*). Cell lysates were prepared from both cell lines as samples of GPI–GnT with or without ARV1 and an acceptor substrate PI. These cell lysates were then incubated with UDP-[^3^H]GlcNAc as a donor substrate to generate and visualize the early GPI biosynthetic intermediates. Both lysates produced [^3^H]GlcNAc-PI and [^3^H]GlcN-PI, the first and second step products, using endogenous PI. The generated GPI biosynthetic intermediates were analyzed by high-performance thin-layer chromatography. The intensities of [^3^H]GlcNAc-PI and [^3^H]GlcN-PI spots from ARV1-rescued lysate were more than twice than those from the empty vector-transfected one (Fig. 4*B*). When radioisotope intensities were normalized by the intensities of phosphatidylcholine in the TLC lanes (Fig. S3), products with ARV1 were nearly five times as intense as those without ARV1 (Fig. 4*B*). These findings showed that the activity of GPI–GnT complex decreased in the absence of ARV1.

### ARV1 enhances usage of PI by GPI–GnT complexes

To study the mechanistic basis of ARV1’s enhancement of the activity of GPI–GnT, a membrane-free enzyme assay using affinity-purified GPI–GnT complexes with or without ARV1 was next established. For this, GPI–GnT complexes bearing GST-PIGA were solubilized in a buffer containing 1% digitonin from cells lacking or expressing FLAG-ARV1 and were captured by glutathione-Sepharose. Washed beads bearing GPI–GnT complexes with or without FLAG-ARV1 were incubated with a constant amount of UDP-[^3^H]GlcNAc and different concentrations of bovine liver PI (0, 10, and 100 μM). Generated [^3^H]GlcNAc-PI amounts were analyzed by high-performance thin-layer chromatography and quantified (Fig. 4*C*). Without adding bovine PI, ARV1-containing GPI–GnT complexes, but not ARV1-less GPI–GnT complexes, generated a clearly detectable amount of [^3^H]GlcNAc-PI. Therefore, the isolated ARV1-containing GPI–GnT complexes carried PI available for the reaction. With increased doses of bovine PI, both ARV1-containing and ARV1-less GPI–GnT complexes generated increased amounts of [^3^H]GlcNAc-PI. At 100 μM bovine PI, ARV1-containing GPI–GnT generated at least five times as much [^3^H]GlcNAc-PI as ARV1-less GPI–GnT did (Fig. 4*C*). These results indicate that ARV1 confers the GPI–GnT complex with the ability to retain PI and to use it more efficiently.

### ARV1 enhanced production of GlcNAc–PI by GPI–GnT in live cells

**Figure 5:**
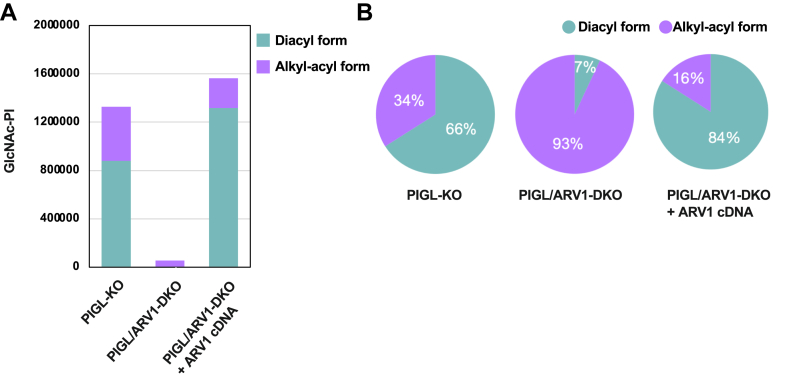
**Fewer GPI intermediates are produced without ARV1.***A*, total amounts of GlcNAc-PI with alkyl/acyl PIs (*purple*) or diacyl PIs (*indigo*) in PIGL-KO HEK293 cells and PIGL/ARV1-DKO HEK293 cells without or with ARV1 rescue. *B*, pie charts showing the percentage of two forms of PIs based on (*A*). *Indigo*, diacyl form of GlcNAc-PI; *purple*, alkyl-acyl form of GlcNAc-PI. (*A* and *B*) show representative data of two repeated lipidomic analyses.

To directly compare the production levels of GlcNAc–PI in ARV1-sufficient and ARV1-KO live cells, PIGL-KO and PIGL/ARV1-DKO HEK293 cells were used. In addition, PIGL/ARV1-DKO HEK293 cells were permanently transfected with an ARV1-expressing vector or an empty vector to evaluate the effects of ARV1 rescue. PIGL, the de-N-acetylase involved in the second step of GPI biosynthesis, converts GlcNAc–PI to GlcN–PI. Cells lacking PIGL accumulate GlcNAc–PI, allowing mass spectrometric quantitation of GlcNAc–PI species with various hydrocarbon chain compositions in their PI moiety. GlcNAc–PIs with various PI species were generated in these PIGL-defective cells (Table S1). The total amount of GlcNAc–PIs in empty-vector–transfected PIGL/ARV1-DKO cells was only approximately 4% of that in PIGL-KO cells (Fig. 5*A*). The expression of ARV1 in PIGL/ARV1-DKO cells restored the total GlcNAc–PI amount to approximately the same level as in PIGL-KO cells (Fig. 5*A*). This confirmed that ARV1 is required for the efficient action of GPI–GnT in live cells. In addition, limited species of PI, mainly alkyl/acyl species (Fig. S3), were used in the absence of ARV1 (Fig. 5*B* and Table S1), suggesting that ARV1 enables GPI–GnT to use a wider variety of PI species, especially diacyl PI.

### Structural prediction of GPI–GnT complex by AlphaFold3

**Figure 6:**
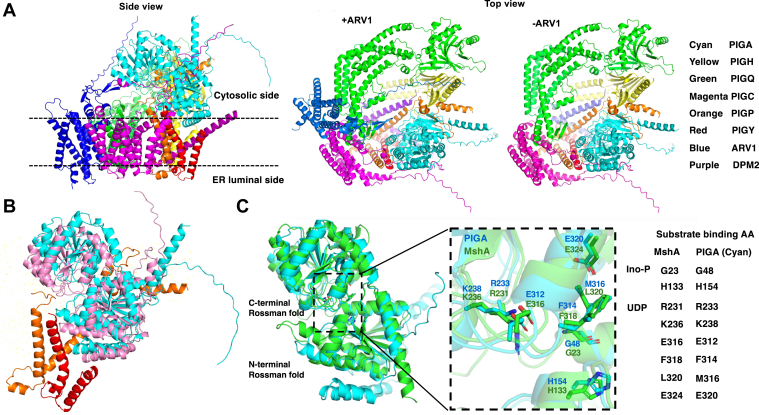
**Structure of GPI-GnT complex predicted by Alphafold3.***A*, side view (*left*) and top view (*middle*) of the full complex, and top view of an ARV1-less complex (*right*). Location of the ER membrane with cytosolic and luminal orientation are indicated in the side view. *Cyan*, PIGA; *yellow*, PIGH; *green*, PIGQ; *magenta*, PIGC; *orange*, PIGP; *red*, PIGY; *blue*, ARV1; and *purple*, DPM2. *B*, the orientation of PIGA within the GPI–GnT complex with (*cyan*) or without (*pink*) PIGY (*red*) and PIGP (*orange*). *C*, the overlaid structures of MshA (*green*) and PIGA (*cyan*). Substrate-binding region is expanded with relevant amino acid side chains of PIGA (*top*) and MshA (*bottom*) indicated. Conserved amino acids (AA) involved in substrate binding are aligned on the *right*. Ino-P, inositol-phosphate.

Recently, AlphaFold3 has become available as a tool for the structural prediction of complexes consisting of multiple proteins. Eight components of GPI–GnT complex, namely, PIGA, PIGC, PIGH, PIGP, PIGQ, PIGY, DPM2, and ARV1, were input into AlphaFold3. The top ranked model as shown by PyMOL software is presented in Figure 6*A*. In this model, the complex consists of a membrane-embedded portion and a cytoplasmic portion with a very small luminal portion (side view on the left). The membrane-embedded portion is made up with almost entire parts of PIGC (magenta), PIGY (red), DPM2 (purple) and ARV1 (blue), a major part of PIGQ (green), and parts of PIGH (yellow) and PIGP (orange), whereas the cytoplasmic portion is made up with almost entire part of PIGA (cyan) and parts of PIGH, PIGP, and PIGQ. Looking from the top (middle), the membrane-embedded portion is organized like a C-shaped partial ring with a big central hole and an opening between PIGQ and PIGH. The partial ring is closed in the cytoplasmic portion by association of PIGQ with PIGH (top view). In the complex, PIGQ associates with ARV1 and PIGC on its C-terminal part and with PIGH on its N-terminal part (top view); and PIGC and PIGH associate with PIGA (side view). In this model, PIGA does not have a transmembrane helix and is peripherally associated with the cytoplasmic side of the membrane (side view), being located on a part containing PIGP, PIGY, and DPM2 of the membrane-embedded partial ring (top view). Thus, these eight proteins form a closed ring with membrane-embedded and cytoplasmic portions.

Analyzed with SUPERPOSE in CCP4 software (35), in the absence of ARV1, there was no significant structural change in other components (Fig. 6*A*, compare middle and right), suggesting a lack of structural role of ARV1 in the formation of the GPI–GnT complex. Because the modeled complexes do not include PI and UDP-GlcNAc, it is still possible that two complexes might be structurally different in the presence of these substrates.

PIGP, PIGY, and DPM2, all of which have a V-shaped transmembrane domain consisting of two transmembrane helices, associate with PIGA (Fig. 6*A* side view). To determine their importance for the orientation of PIGA in the complex, complexes lacking one of them were modeled. Whereas removal of DPM2 did not affect orientation of PIGA, removal of PIGP or PIGY affected it. In Figure 6*B*, PIGA with PIGP and PIGY in the full complex is overlaid with PIGA in the complex lacking PIGP and PIGY. Orientation of PIGA is clearly changed in the absence of PIGP and PIGY, indicating that PIGP and PIGY, but not DPM2, are necessary to maintain PIGA’s orientation within GPI–GnT complex (Fig. 6*B*).

PIGA belongs to the GT 4 family having GT-B folds. The modelled PIGA structure is most like crystal structure of *Corynebacterium* MshA (Fig. 6*C*). MshA catalyzes a transfer of GlcNAc from UDG-GlcNAc to inositol phosphate, a reaction very similar to the PIGA-mediated transfer of GlcNAc to PI (36). The binding sites of UDP and inositol phosphate in MshA were determined (36). Amino acids in the C-terminal Rossman fold of MshA that are involved in UDP binding (R231, K236, E316, F318, L320, and E324) were well conserved in PIGA (R233, K238, E312, F314, M316, and E320). Critical amino acids in the inositol phosphate–binding site near the hinge region of the MshA N-terminal domain (G23 and H133) were also well conserved in PIGA (G48 and H154). From these results, we propose that like many other GT-B type glycosyltransferases, PIGA catalyzes GlcNAc–PI formation using a donor binding site in the C-terminal Rossman fold and an acceptor binding site in the N-terminal Rossman fold facing to each other. However, this predicted inositol phosphate–binding site of PIGA is located approximately 24 Å above the membrane, suggesting a presence of some mechanism to properly present inositol phosphate moiety in PI to the binding site. The ring formation of the enzyme complex must thus have some functional importance.

## Discussion

In this study, we demonstrate that GPI–GnT requires ARV1 to efficiently generate GlcNAc–PI both in cell lysates and in live cells. ARV1 associates with the GPI–GnT complex *via* binding to PIGQ, and the physical association of ARV1 with PIGQ is essential for the enhancement of GPI–GnT activity by ARV1. Taking these findings together with a recent report showing that isolated trypanosomal GPI–GnT contained ARV1 (30), we propose that ARV1 is a component of GPI–GnT in mammalian cells and trypanosomes.

It was predicted that yeast ARV1 associates with GPI1, the yeast homolog of PIGQ (29). The modeled complex of yeast ARV1 and GPI1 was highly similar to that of human ARV1 and PIGQ, suggesting that yeast ARV1 also contributes to GPI–GnT. Meanwhile, previous studies in yeast indicated that yeast ARV1 is required for the efficient generation of mannosyl-GlcN-acylPI, the fourth intermediate in GPI synthesis, and proposed that ARV1 is involved in the efficient usage of GlcN-acylPI, the third intermediate, presumably by facilitating the flipping of GlcN-acylPI from the cytoplasmic side to the luminal side of the ER (18, 37, 38). Further studies are required to characterize this possible role of yeast ARV1.

Although the loss of ARV1 substantially reduced the generation of GlcNAc–PI, single KO of ARV1 in HEK293 cells did not reduce cell surface levels of GPI-APs (*e.g.*, CD59, DAF, and FLAER staining), suggesting that the amount of GlcNAc–PI synthesized without the help of ARV1 must be sufficient to support WT levels of GPI-APs on the surface of HEK293 cells. This interpretation must be true because when HEK293 cells knocked in with a hypomorphic PIGA R119W variant were used, knockout of ARV1 resulted in a marked reduction in GPI-APs’ levels (Fig. 1*B*). In contrast to HEK293 cells, single KO of ARV1 in fibroblasts caused clear decreases in the cell surface levels of CD73 and CD109, being consistent with reports on decreased GPI-AP levels on fibroblasts and neutrophils derived from patients with the inherited ARV1 deficiency EIEE38 (27, 28). These results indicate that the levels of GlcNAc–PI required for normal expression of various GPI-APs differ among cell types. It seems that the reduction of certain GPI-APs in brain cells is causative of the neuronal symptoms of EIEE38.

In ARV1-KO human fibroblasts, the cell surface level of CD59 was normal whereas those of CD73 and CD109 were clearly reduced. It is well known that when the amount of available GPI decreased, cell surface levels of various GPI-APs are differently affected (39, 40, 41). The key factor for this variation seems to be strength of efficiency of the C-terminal GPI attachment signal peptides of the proproteins. Proproteins with strong signal peptides, such as CD59 and DAF, efficiently use GPI whereas those with weak signal peptides, such as CD73 and CD109, do not fully obtain GPI, resulting in reduction in their cell surface expression levels.

To date, 10 ARV1 variants were found in 26 ARV1-deficient patients from 14 families (25, 26, 27, 28, 37, 38). All of the patients suffered from seizures and intellectual disability (ID). Some of the variants are shown in Fig. S1*C*. Although those variants are not located in the region for interaction with PIGQ, they might have impacts on function and structure of ARV1 and patients’ symptoms. Patients with missense mutations, Cys34Tyr or Cys61Tyr, both located on the cytosolic side, exhibited ID and hypotonia with controllable seizures (27). These cysteines are conserved in yeast Arv1, are located within the AHG, and form a putative zinc-binding motif together with two other conserved cysteines, Cys 37 and Cys 58, suggesting that this motif may be important for interactions with other molecules such as lipids or proteins (13) (Fig. S1, *C* and *D*). The Gly189Arg mutation is located in the transmembrane region and the Arg residue is likely to disturb the α-helix structure. The Gly189Arg homozygous patients exhibited severe ID, interactable seizures, ataxia, and cardiomyopathy (26, 38, 42). A splicing variant, p.Lys59_Asn98del likely results in the loss of the binding sites with PIGQ. Homozygous patients with this variant presented intractable seizures, ID, and ophthalmological anomalies (25, 28). Another splicing variant, Thr226_Phe271del also led to homozygous patients presenting with ID, seizures, cortical and cerebellar atrophy, delayed myelination, dysmorphic facial features, deafness, and ophthalmological anomalies (28). A frame shift mutation, Ser122Glnfs resulted in the loss of all transmembrane domains, likely leading to a null function protein. The homozygous patient exhibited severe ID, interactable seizures, dysmorphic facial features, finger abnormalities, and abnormal brain MRI (including delayed myelination, T2 hyperintensity with restricted diffusion in the dorsal midbrain, and cerebellar atrophy) (27).

In the *in vitro* enzyme assay using purified GPI–GnT complex, UDP-[^3^H]GlcNAc and bovine PI, ARV1-containing complex, but not ARV1-less complex, generated [^3^H]GlcNAc-PI without the addition of bovine PI. Therefore, PI was retained in the GPI–GnT complex during membrane solubilization in 1% digitonin and affinity-capture to glutathione-Sepharose. The addition of bovine PI at 10 μM to ARV1-containing complex only slightly increased [^3^H]GlcNAc-PI production, whereas 100 μM bovine PI caused a greater increase. Upon the addition of bovine PI to purified GPI–GnT complexes, higher amounts of [^3^H]GlcNAc-PI were generated in the presence of ARV1. These results indicate that ARV1 enabled the GPI–GnT complex to use PI with higher efficiency.

More efficient generation of GlcNAc–PI by GPI–GnT in the presence of ARV1 was also found in live cells. By mass spectrometric analysis of glycolipids from PIGL-KO HEK293 cells with or without ARV1 expression, we quantified various species of GlcNAc-PIs accumulated in these cells and determined the total amounts of GlcNAc-PIs. PIGL-KO cells with ARV1 contained 50 times as much GlcNAc-PIs as PIGL-ARV1 DKO cells did, being consistent with the *in vitro* enzyme assay results and further proving that a lack of ARV1 substantially reduces the initiation of GPI biosynthesis.

Concerning the structures of PI species used by GPI–GnT, we recently found that PIs in HEK293 cells contained 4% to 5% of 1-alkyl, 2-acyl species and that 1-alkyl, 2-acyl PIs are more efficiently used by GPI–GnT than diacyl PIs are in making GlcNAc-PI (Li, X.Y. *et al.*, manuscript in preparation). Overall, 34% of GlcNAc–PIs in ARV1-expressing PIGL-KO HEK293 cells were of the 1-alkyl, 2-acyl form (Fig. 5*B*), confirming that the GPI–GnT preferentially use 1-alkyl, 2-acyl PI. In contrast, although the total amount was much smaller, 93% of GlcNAc–PI in ARV1-lacking PIGL-KO cells was of the 1-alkyl, 2-acyl form (Fig. 5, *A* and *B*), indicating that ARV1-less GPI–GnT preferentially uses 1-alkyl, 2-acyl PI. Therefore, GPI–GnT requires ARV1 to efficiently use both diacyl and 1-alkyl, 2-acyl forms of PI. It seems that ARV1, as a component of GPI–GnT enzyme complex, presents both forms of PI as substrates to the enzyme and that catalytic site of GPI–GnT has higher affinity to 1-alkyl, 2-acyl PI than to diacyl PI, leading to increased % in 1-alkyl, 2-acyl GlcNAc–PI.

Computational analysis using AlphaFold3 revealed that GPI–GnT was ring-shaped. The predicted inositol phosphate–binding site of PIGA was located far above the membrane, and the distance to the transmembrane domain was approximately 24.2 Å, which appears to be longer than PI. In *Corynebacterium* MshA, which like PIGA also belongs to the glycosyltransferase family 4, it was reported that conformational change occurred when the donor substrate UDP-GlcNAc bound and it rotated 90° around the axis connecting the N-terminal and C-terminal domains (36). It is possible that PIGA also changes the conformation when the donor binds. What is the functional meaning of the ring formation and what is the function of other factors? We speculate that a special ER membrane domain, enriched with PI, is formed inside the ring for the efficient biosynthesis of GPI, and ARV1 functions to transfer PI into the ring.

ARV1 was originally identified as a regulator of sterol metabolism more than 20 years ago and has been studied by several research groups as a regulator of the homeostasis of various lipids in yeast and mammals (14). However, the mechanistic basis of its function has remained poorly understood. ARV1 is located in the ER in mammalian cells (22), while it is expressed in both the ER and the Golgi apparatus in yeast (16). Because GPI–GnT exists exclusively in the ER, Golgi-resident yeast ARV1 must exist as a free form or together with other partner protein(s) working in regulating lipid homeostasis. It is also possible that a similar situation prevails for some of the ER-resident ARV1. Indeed, CaARV1, ARV1 of the yeast *C. albicans*, associates with and stabilizes lanosterol 14-α-demethylase (CaErg11), the rate-limiting enzyme in sterol biosynthesis. It was reported that *C. albicans* cells lost virulence and became azole-hypersusceptible when CaARV1 was absent (21). Therefore, physical association with key proteins in various lipid biosynthetic pathways is one mechanism by which ARV1 functions.

## Experimental procedures

### Generation of KO, KI, and their rescued cells

HEK293 cells (ATCC CRL-1573), human dermal fibroblasts (ATCC), and their KO or KI derivatives were cultured in DMEM/F-12 (Nacalai Tesque) with 10% heat-inactivated fetal bovine serum (172012-500 ml; Sigma). These cell lines were maintained in an incubator at 37 °C and 5% CO_2_. The cell lines used in this study are listed in Table S2.

ARV1-KO human fibroblasts were generated using a lentiviral CRISPR-Cas9 system. Four oligonucleotides encoding gRNAs (sgRNAs, Table S3) targeting the first exon of *ARV1* were synthesized and annealed and then integrated into LentiCRISPRv2-puro (Addgene) between BsmbI-digested sites. LentiCRISPRv2-puro-ARV1-KO1 and LentiCRISPRv2-puro-ARV1-KO2 were transiently transfected into LentiX 293T cells (Clontech, cultured in DMEM with 10% FBS) together with pVSVG, pLC1, and pLC2 (Thermo Fisher Scientific) by PEI-Max (Polyscience) to produce lentivirus. Viral medium was collected 2 days after lipofection and filtered through a Millex-HV syringe filter (SLHV003R, Merck Millipore) to remove cell debris. Filtered viral medium was mixed with 8 μg/ml polybrene (Sigma Aldrich) and added to the target cells. Cells bearing DNAs transduced by lentivirus were maintained in a medium containing 1 μg/ml puromycin for 2 weeks, during which WT cells died. The puromycin-treated cells were used as bulk KO cells.

To establish the rescued cells, KO cells were transduced with lentivirus expressing a hygromycin resistance gene and *ARV1* bearing silent base substitution mutations to confer resistance against Cas9 cleavage and were maintained in a medium containing 100 μg/ml hygromycin.

PIGAKI #6, PIGOKI #49-2, and PIGTKI #64 HEK293 cells were established using CRISPR/Cas9-mediated KI. In brief, HEK293 cells were cotransfected with pX330-mEGFP generated from pX330-U6-Chimeric_BB-CBh-hSpCas9 (Addgene) containing a target gRNA sequence (TTGCTCAGGTACATATTTGT for PIGA, CAACCTGAGATCGGTAGAGC for PIGO, and TCGGTGCAGACCACCTCCCG for PIGT) and a donor pBS plasmid containing a 1.8 kb homologous region (for efficient target recombination). This region includes a pathogenic mutation (PIGA, c.355C>T; PIGO, c.355C>T; PIGT, c.547A>C) and silent base substitution mutations to confer resistance against Cas9 cleavage and to generate a restriction enzyme for easy genotyping (AflII site for PIGA, SalI site for PIGO, AvrII site for PIGT). After initial sorting of the EGFP-positive cells followed by further sorting of the population showing decreased CD59 expression, cell clones with successful KI were picked up and validated by Sanger sequencing.

ARV1 was further knocked out in these three KI clones to generate PIGA^R119W^KI/ARV1-KO, PIGO^R119W^KI/ARV1-KO, and PIGT^T183P^KI/ARV1-KO. pX330-mEGFP vectors containing ARV1 targeting gRNAs, KO1 and KO2, were transiently transfected into each KI cell. Two days after lipofection, GFP-positive cells were sorted and their culture was continued for 2 weeks, after which they were used as bulk DKO cells.

To establish ARV1-rescued cells from PIGA^R119W^KI/ARV1-KO cells, FLAG-ARV1 was expressed using the tet-On system (Clontech). PLAT-GP retroviral packaging cells were cotransfected with pRetro-Tet-On Advanced, pRetro-Tight-Pur FLAG-ARV1, and pVSVG using PEI-Max transfection reagent and cultured at 37 °C overnight. Cells were incubated in fresh medium at 32 °C for 1 day to produce retrovirus. Viral medium was harvested and filtered by membrane filters and then added to HEK293 PIGA-KI/ARV1-KO cells, followed by centrifugation at 3500 rpm and 32 °C for 2 h. After 12 h of culture, the medium was replaced with a fresh one, and culture of the cells was continued at 37 °C for 2 more days. Finally, the cells were maintained in a medium with 600 μg/ml G418 (InvivoGen) and 1 μg/ml puromycin for 2 weeks to obtain complete antibiotic-resistant cells for further use. To induce the expression of FLAG-ARV1, 0.5 μg/ml doxycycline was added to the medium and incubated for 24 h. The expression of FLAG-ARV1 protein was detected by Western blotting with anti-DYKDDDDK tag antibody (Wako Chemicals).

PIGA-KO, PIGQ-KO, ARV1-KO, PIGA/ARV1-DKO, and PIGL/ARV1-DKO cells were generated in the lab using the CRISPR-Cas9 system. gRNA targeting oligonucleotide sequences are shown in Table S2.

### Flow cytometry

Cells were harvested using Trypsin/EDTA or 5 mM EDTA/PBS buffer and washed using cold PBS twice. To detect specific cell surface GPI-APs, cells were stained with anti-CD59 mAb (clone 5H8), anti-CD55/DAF mAb (clone IA10), phycoerythrin (PE)-labeled anti-human CD73 (clone AD2, BioLegend), and PE-labeled anti-human CD109 (clone W7C5, BioLegend) in FACS buffer (PBS containing 1% BSA and 0.1% NaN_3_) for 25 min on ice. After washing with FACS buffer three times, cells were stained with PE-labeled anti-mouse IgG (clone poly4053, BioLegend) in FACS buffer for 20 min on ice. To measure the total GPI-APs on the cell surface, cells were stained with aerolysin, which binds to the GPI moiety of various GPI-APs, using FLAER (CEDARLANE) in FACS buffer for 20 min on ice. The cells were then washed using FACS buffer before analysis. Stained cells were analyzed using MACSQuant YVB (Miltenyi Biotec).

### Analysis of protein complex

Immunoprecipitation was used for protein complex analysis. PIGQ-KO HEK293 cells were plated in a 15-cm dish and, after overnight culture, were transiently transfected with vectors bearing 3HA-ARV1, FLAG-PIGA, FLAG-PIGH, FLAG-PIGC, FLAG-PIGY, and FLAG-PIGP. At the same time, GST-PIGQ or empty vector was also transiently expressed in the cells. After the cells were harvested and washed in cold PBS twice, they were lysed in 1 ml of 1% digitonin lysis buffer [25 mM Hepes pH7.4, 150 mM NaCl, 1% digitonin (Wako Chemicals), and cOmplete protease inhibitor (Roche)] with rotation for 20 min in a cold room. The cell lysates were centrifuged at 21,900*g* for 15 min to remove cell debris. The supernatant was divided into three (3:1:1) and transferred to three new tubes containing 60 μl of rat anti-HA beads (Sigma), 20 μl of anti-FLAG beads (Sigma), and 20 μl of glutathione beads (Cytiva), respectively. Anti-HA beads were used to capture HA-ARV1–containing protein complexes and the two other beads were used to determine expression level of FLAG-tagged and GST-tagged proteins (Input). The beads and cell lysate mixture were rotated in a cold room for 1 h, after which the beads were collected and washed with lysis buffer three times. The protein complex attached to the beads was eluted by adding 2×sample buffer containing 5% β-mercaptoethanol.

PIGA-KO HEK293 cells were plated in a 10-cm dish, and empty vector or vectors bearing 3HA-ARV1, GST-PIGQ, FLAG-PIGH, FLAG-PIGC, FLAG-PIGY, FLAG-PIGP, and FLAG-PIGA were transiently expressed in the cells. In contrast to the procedure for PIGQ-KO HEK293 cells, glutathione beads were used to capture the protein complex. The other steps were the same as described above. The prepared protein samples were separated using a 7.5%–15% ExtraPAGE One Precast Gel (Nacalai Tesque) and analyzed by Western blotting. In brief, the proteins in the gel were transferred to a polyvinylidene fluoride membrane (Merck Millipore) for immunoblotting. This membrane was blocked in 5% nonfat milk/TBS-T (10 mM Tris-NaOH pH8.0, 150 mM NaCl, 0.1% Tween-20) at room temperature (RT) for 1 h or in a cold room overnight. The membrane was then cut into pieces to detect the different proteins and incubated with primary antibodies in Can Get Signal Solution 1 (TOYOBO) at RT for 1 h or in a cold room overnight. After three washes with TBST, the membranes were incubated with horseradish peroxidase–labeled secondary antibodies in Can Get Signal Solution 2 (TOYOBO) for 45 min at RT. Finally, the membranes were washed three times with TBST and exposed to Amersham ECL Prime Western Blotting Detection Reagent (Cytiva). Images were taken using ImageQuant LAS 4000 Mini (GE Healthcare).

### AlphaFold2 prediction of ARV1 and PIGQ association sites

To predict the sites of association between hARV1 and hPIGQ, the full sequences of hARV1 (UniProtKB = Q9H2C2) and hPIGQ (UniProtKB = Q9BRB3) were submitted to AlphaFold2 (ColabFold v1.5.3: AlphaFold2 using MMseqs2) together and analyzed by default codes (43). The models of two proteins with several associated sites were successfully generated. The top ranked model was applied in the following work, and the figures were generated by Open-Source PyMOL 2.3. The model of the association of yARV1 (UniProtKB=Q06541) and yGPI1 (UniProtKB=P53306) was generated by the same method.

### Generation of ARV1 mutants and their functional analysis

Various ARV1 mutants (L53P, Y71A, D72A, D72N, N79Q, N79G, K84A, K84R, ▵53–137) were generated by the mutagenesis of pME-3HA-ARV1 using in-fusion cloning. To analyze the binding of ARV1 mutants and PIGQ, HA-ARV1 mutants and FLAG-PIGQ were transiently overexpressed together in HEK293 cells, and the protein complexes were analyzed by Western blotting after immunoprecipitation by anti-FLAG M2 beads (Sigma-Aldrich), as described above. The intensity of protein bands was quantified using ImageJ (version 2.3.0/1.53q) and the intensity of HA-ARV1 bands was normalized by that of FLAG-PIGQ bands. For the functional analysis, ARV1 mutants were transiently overexpressed in HEK293 PIGA^R119W^KI/ARV1-KO cells to rescue the expression of cell surface GPI-APs, and the expression of CD59 and DAF was determined by flow cytometry. The mean fluorescence intensities of CD59 and DAF were quantified using FlowJo 10.9.0.

### Analysis of GPI–GnT activity *in vitro*

The method of preparing GPI–GnT protein complex was as described previously (9). In this case, PIGA/ARV1-DKO HEK293 cells were used in protein complex production to avoid endogenous PIGA or ARV1 protein. The PIGA/ARV1-DKO cell line was stably transfected with pMEEBO GST-PIGA by hygromycin (100 μg/ml) selection. Then, the bulk cells with restored expression of GPI-APs were stained with anti-CD59 mAb and PE-labeled anti-mouse IgG in Hanks’ Balanced Salt Solution (H6648) and sorted by FACSAria (BD) to collect cells positively stained for CD59 mAb. ARV1 was rescued in this cell line by tet-On system-controlled expression of FLAG-ARV1 mentioned above, while empty vector pRetro-Tight-Pur was used as a null control. Both cell lines were maintained in a medium with 100 μg/ml hygromycin, 600 μg/ml G418, and 1 μg/ml puromycin.

To prepare cell lysate, 0.5 μg/ml doxycycline was added to the medium 1 day before harvesting, and 5 μg/ml tunicamycin (Wako Chemicals) was added 3 h before harvesting. A total of 10^7^ cells were prepared for each test. After washing twice with iced PBS, cells were resuspended in 500 μl of cold buffer (20 mM Hepes-NaOH pH 7.4, cOmplete protease inhibitor EDTA-free) and kept on ice for 5 min, after which 500 μl of iced hypotonic buffer (20 mM Hepes-NaOH pH 7.4, cOmplete protease inhibitor EDTA-free, 500 mM sucrose) was added. After homogenization using a Dounce tissue grinder (Wheaton) 30 times on ice, the mixture was centrifuged for 10 min at 2500 rpm, after which the supernatant was collected and subjected to ultracentrifugation at 100,000*g* for 1 h at 4 °C. The pellet obtained after centrifugation contained the protein for reaction. To prepare purified protein complex, the cells (10^7^ cells for each test) were lysed in 1% digitonin lysis buffer (50 mM Hepes-NaOH pH 7.4, 25 mM KCl, 5 mM MgCl_2_, cOmplete protease inhibitor EDTA-free, 1% digitonin) by rotating in a cold room for 20 min, after which insoluble materials were removed by centrifugation. Protein complexes with GST-PIGA were collected using prewashed glutathione-Sepharose 4B. The protein complexes bearing glutathione beads were washed with cold PBS three times.

Cell lysates and the glutathione beads with protein complex were incubated with 0.2 μM UDP-[^3^H]GlcNAc (14 μCi/ml; American Radiolabeled Chemicals) for 1 h at 37 °C in reaction buffer (50 mM Hepes-NaOH pH 7.4, 25 mM KCl, 5 mM MgCl_2_, 5 mM MnCl_2_, cOmplete protease inhibitor EDTA-free, 0.2 μg/ml tunicamycin, 1 mM ATP, 0.5 mM DTT). To test additional PI substrate dependence of the purified protein complex, bovine liver PI (Sigma-Aldrich) dissolved in 1% digitonin was added. Products of the reaction were extracted twice with water-saturated *n*-butanol, followed by evaporation with a vacuum centrifuge. The dried materials were dissolved in chloroform/methanol = 1:1 (v:v) and separated by thin-layer chromatography on Kieselgel 60 (Merck) with a development solution of chloroform/methanol/1 M NH_4_OH = 10:10:3 (v:v:v). The radioactive products [^3^H]GlcNAc-PI and [^3^H]GlcN-PI were detected by phosphorimaging using ImageAnalyzer BAS 1500 (Fuji Film Co). Data were analyzed by ImageJ (version 2.3.0/1.53q).

### Lipidomics of KO cells

Cell pellets (10^6^ cells) of each of PIGL-KO HEK293 and PIGL/ARV1-DKO HEK293, rescued with ARV1 or an empty vector using the tet-On system mentioned above, were sent to Kazusa DNA Institute for the analysis of GPI intermediates by mass spectrometry using an unbiased lipidomic method.

Untargeted lipidomics was performed as reported previously with some modifications (44, 45). Briefly, the frozen cell pellets were redissolved in 200 μl of methanol (Wako Chemicals) containing EquiSPLASH (Avanti Polar Lipids) as an internal standard. After sonication for 30 s and vortexing for 2 s, 160 μl of the suspension was collected and 80 μl of chloroform (Wako Chemicals) was added and then vigorously agitated at 750 rpm for 20 min at 20 °C. Subsequently, 16 μl of pure water (Wako Chemicals) was added and vigorously agitated at 750 rpm for 20 min at 20 °C. The supernatant was collected by centrifugation at 1670*g* for 10 min at 20 °C and transferred to an LC vial.

LC-MS/MS analysis was carried out using quadrupole time-of-flight/MS (TripleTOF 6600; SCIEX) coupled with an ACQUITY UPLC system (Waters), as previously reported (46). The LC separation was performed with gradient elution of mobile phase A [methanol/acetonitrile/water (1:1:3, v/v/v) containing 5 mM ammonium acetate (Wako Chemicals) and 10 nM EDTA (Dojindo)] and mobile phase B [isopropanol (Wako Chemicals) containing 5 mM ammonium acetate and 10 nM EDTA]. The flow rate was 300 μl/min at 45 °C using an L-column3 C18 (50 × 2.0 mm i.d., particle size 2.0 μm; Chemicals Evaluation and Research Institute). The solvent composition started at 100% (A) for the first 1 min and was changed linearly to 64% (B) at 7.5 min, where it was held for 4.5 min. The gradient was increased linearly to 82.5% (B) at 12.5 min, followed by 85% (B) at 19 min, 95% (B) at 20 min, 100% (A) at 20.1 min, and 100% (A) at 25 min.

The raw data files from quadrupole time-of-flight/MS were converted to MGF files using the program SCIEX MS converter for quantitative analysis with 2DICAL (Mitsui Knowledge Industry). Identification of the molecular species was accomplished by comparison with retention times and MS/MS spectral data from the information-dependent acquisition mode.

### AlphaFold3 prediction of GPI–GnT complex

To predict the structure of the GPI–GnT complex, the full sequences of hPIGA (UniProtKB = P37287), hPIGH (UniProtKB = Q14442), hPIGC (UniProtKB = Q92535), hPIGQ (UniProtKB = Q9BRB3), hPIGP (UniProtKB=P57054–2), hPIGY (UniProtKB = Q3MUY2), hDPM2 (UniProtKB = O94777), and hARV1 (UniProtKB = Q9H2C2) were submitted to the AlphaFold Server (AlphaFold3, Google DeepMind) together and analyzed using default codes (47). The models of GPI–GnT were successfully generated. The top ranked model was applied in subsequent work, and the figures were generated by Open-Source PyMOL 2.3. The structure of *Corynebacterium* MshA (UniProtKB = Q8NTA6) was submitted to the AlphaFold Server and an overlay of PIGA and MshA was generated using PyMOL 2.3.

## Data availability

All data are contained within the manuscript and the supporting information.

## Supporting information

This article contains supporting information (48, 49).

## Conflicts of interest

The authors declare that they have no conflicts of interest with the contents of this article.
