# Supplementary material for: ARV1 is a component of the enzyme initiating glycosylphosphatidylinositol biosynthesis[image]
Source: J Biol Chem. 2025 May 14;301(6):110236. doi: 10.1016/j.jbc.2025.110236 (PMC12182360; doi:10.1016/j.jbc.2025.110236)
Supplement: Supporting Information [file mmc1.pdf]

Supporting Information

Title

ARV1 is a component of the enzyme initiating glycosylphosphatidylinositol biosynthesis

TianTian Lu, Saori Umeshita, Kae Imanishi, Yicheng Wang, Yi-Shi Liu, Masamichi Nagae, Yuya Senoo, Kazutaka Ikeda, Morihisa Fujita, Taroh Kinoshita, and Yoshiko Murakami[#]

The contents

Figures S1-S3
Tables S1-S3

Figure S1

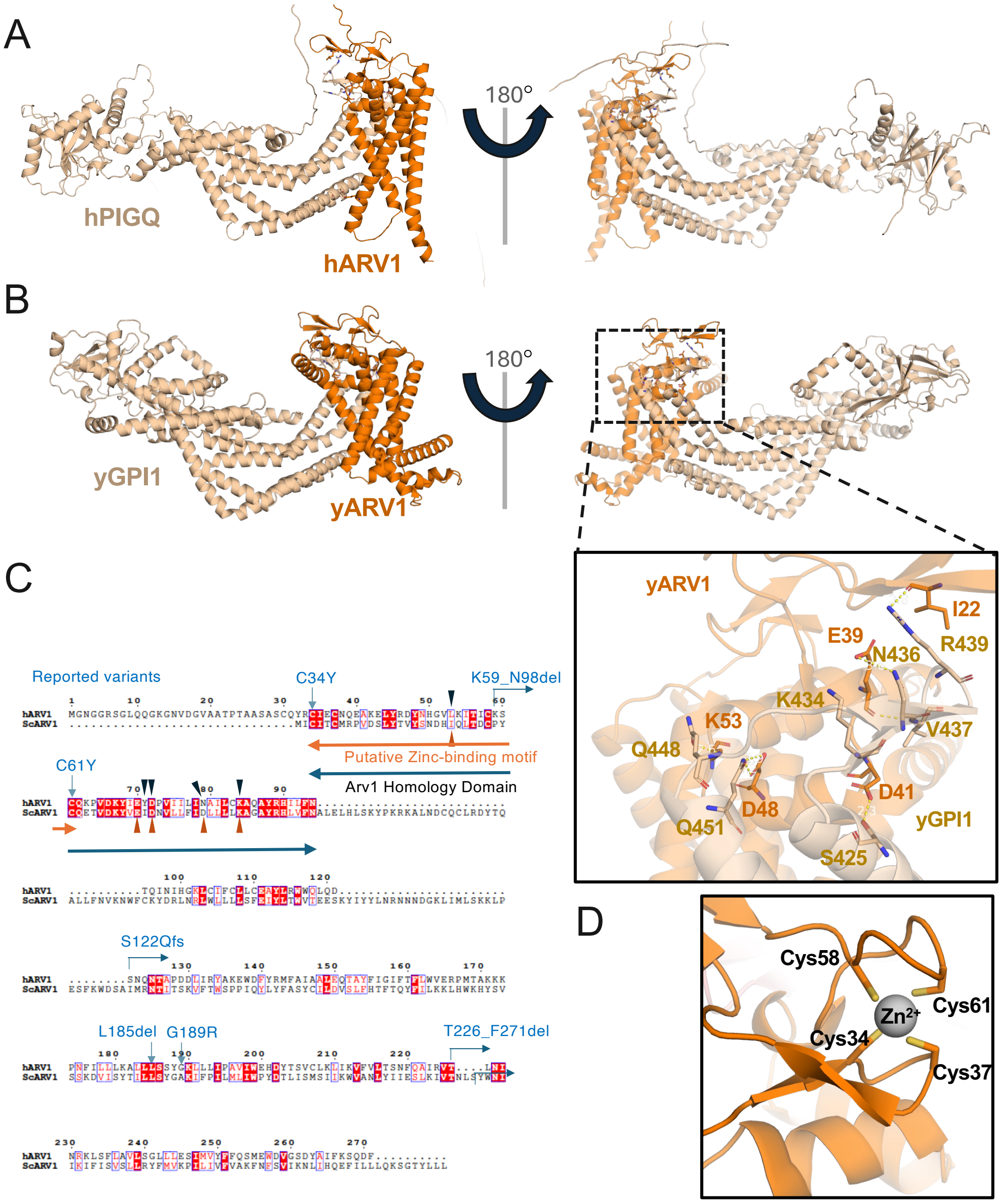

Structural alignment of human and yeast ARV1.

A. AlphaFold2-predicted human ARV1 and PIGQ complex model.

ARV1, orange; PIGQ, khaki. **B.** AlphaFold2-predicted yeast ARV1 and GPI1 complex model. ARV1, orange; GPI1, khaki; oxygen atom, red; nitrogen atom, blue; hydrogen bond, yellow dotted line. The enlarged view shows the amino acids involved in binding. **C.** Alignment of amino acids of human and yeast ARV1. Conserved amino acids are enclosed in red squares. Some of the reported variants from the ARV1 deficient patients are shown in blue. Black arrowhead, the amino acids of hARV1 involved in binding to hPIGQ; brown arrowhead, the amino acids of yARV1 involved in binding to yGPI1. **D.** Putative Zinc-binding motif predicted by Alphafold3

Figure S2

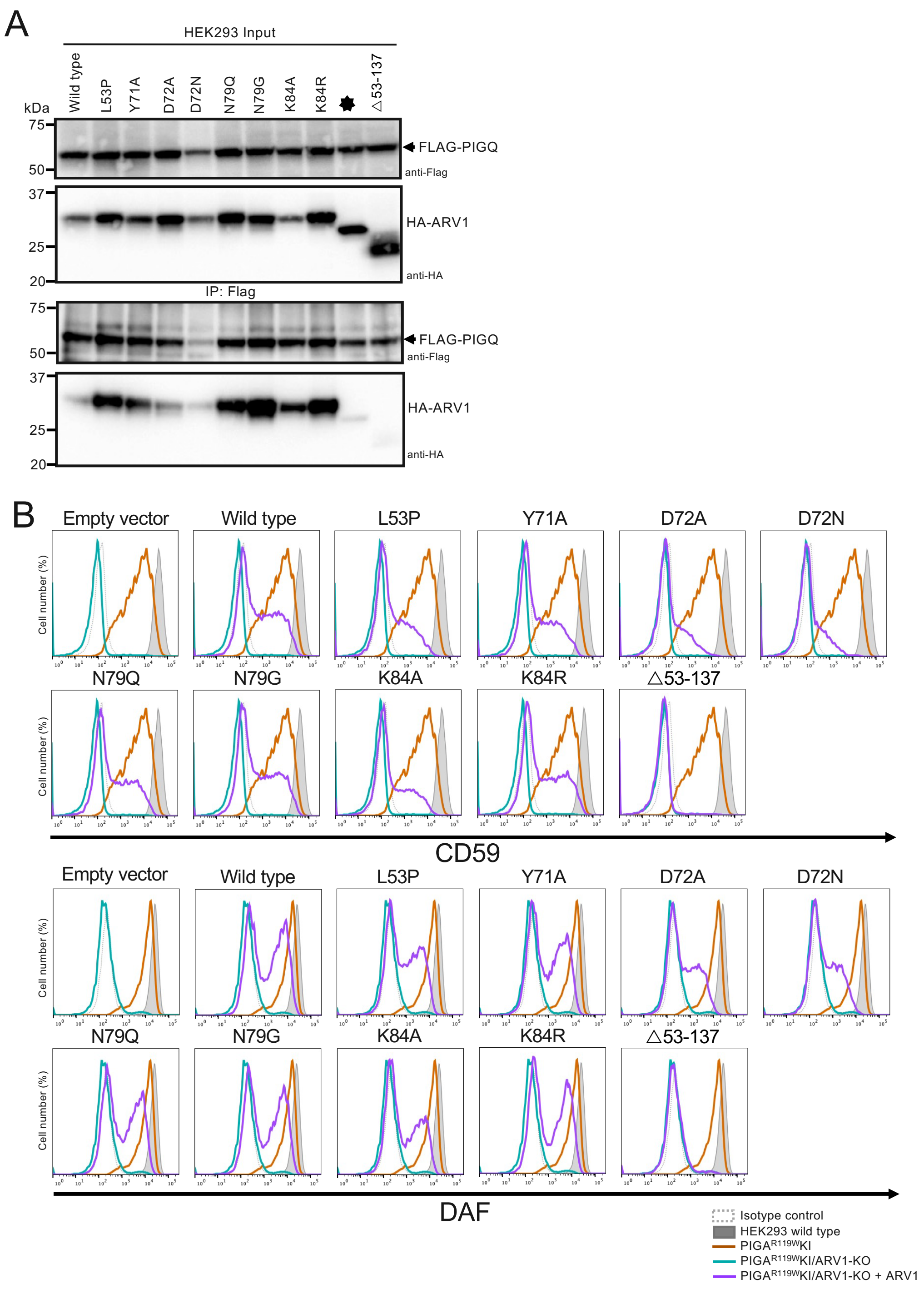

ARV1 mutants modulate GPI-GnT complex activity.

A. Co-IP analysis of FLAG-PIGQ and HA-ARV1 mutants in HEK293 cells. Western blot analysis of quantification data shown in Fig 4C. Septagram-marked mutant is not dealt with in this paper. **B.** FACS analysis of GPI-AP expression in HEK293 PIGA^{R119W}KI/ARV1-KO cells transfected with ARV1 mutants, for quantification data shown in Fig 4D. A and B show representative data of three repeated analyses.

Figure S3

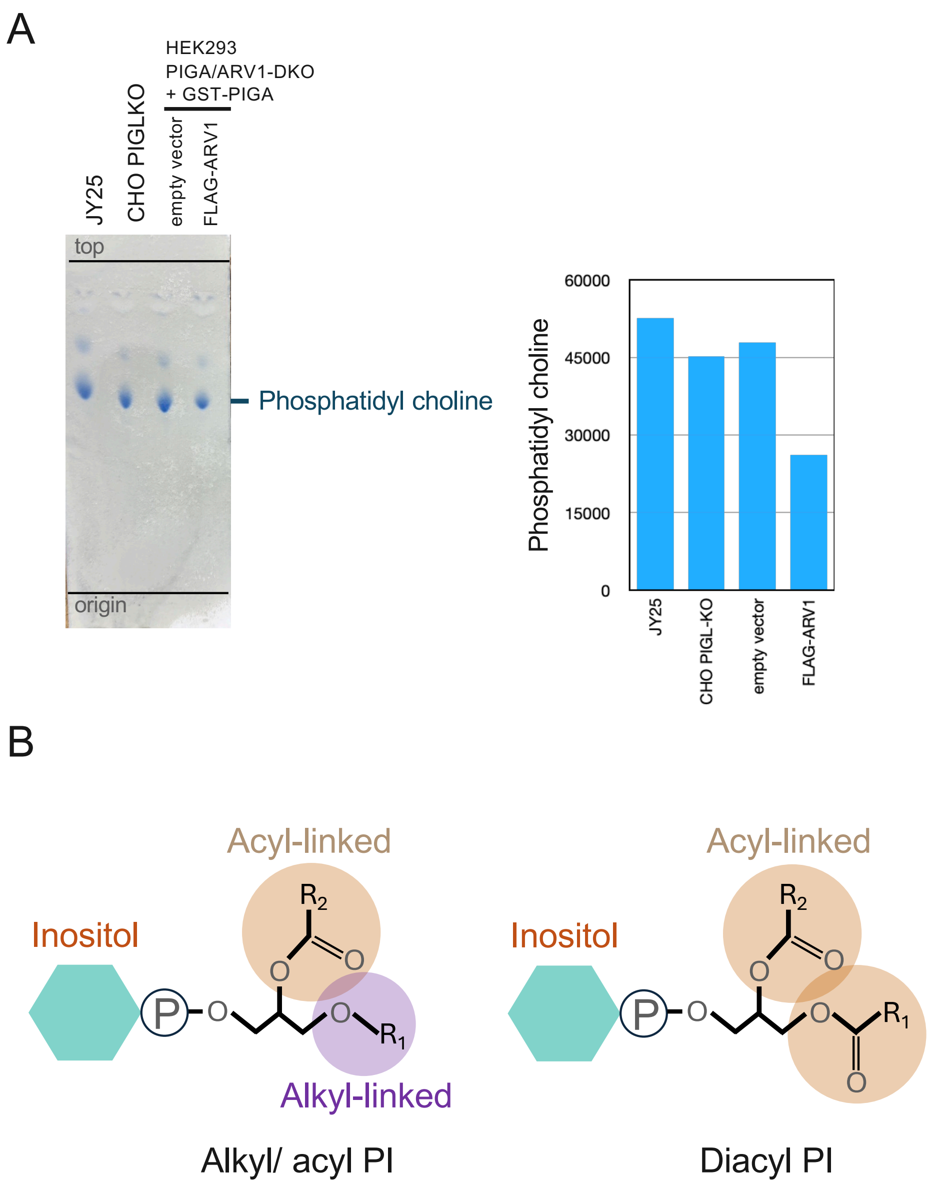

Staining of phosphatidylcholine, as a loading control, and structural difference between alkyl/acyl PI and diacyl PI.

A. Left: Molybdenum blue staining reagent-stained phosphatidylcholine on the HPTLC plate of Fig 4B. Right: Quantification of band intensity. The value of the spots' intensities was gray value measured by ImageJ. Representative data of three repeated experiments. **B.** Schematic of alkyl/acyl PI and diacyl PI structure.

Table S1.
Results of lipidomics analysis of GlcNAc-PI in HEK293 cells

m/z	RT	Lipid species in GlcNAc-PI	PIGLKO	PIGL/ARV1DKO +Empty	PIGL/ARV1DKO +ARV1 rescue
1088.6287	7.51	18:0/20:4	411816	875	604843
1112.6287	7.47	18:0/22:6	140718	2143	229244
1066.6443	7.80	18:0/18:1	80086	88	103461
1070.6181	7.28	16:0e/22:6	79213	22745	49799
1024.6338	7.57	16:0e/18:1,18:1e/16:0	68384	679	53164
998.6181	7.50	16:0e/16:0	64333	0	25951
1114.6443	7.58	18:0/22:5	53232	369	62586
1026.6494	7.97	18:0e/16.0	46858	0	13550
1046.6181	7.32	16:0e/20:4	45818	1701	14762
1090.6443	7.67	18:0/20:3	45411	0	72489
1072.6338	7.37	16:0e/22:5,18:1e/20:4	44740	8921	32266
1038.6130	7.37	16:0/18:1,16:1/18:0	34667	0	46686
1052.6651	8.06	18:0e/18:1,16:0e/20:1	30800	1621	25908
1098.6494	7.69	18:0e/22:6	29419	12859	15774
1086.6130	7.19	18:1/20:4,16:0/22:5	28387	0	51658
1064.6287	7.44	18:1/18:1,16:0/20:2	26122	79	57532
1060.5974	7.13	16:0/20:4	24440	0	32983
1074.6494	7.73	18:0e/20:4	23191	2525	5419
1084.5974	7.10	16:0/22:6	17817	0	30349
996.6025	7.22	16:0e/16:1	15204	0	8609
1092.6600	7.94	18:0/20:2	13816	0	19325
1036.5974	7.10	16:1/18:1	3371	101	7593
Total			1327843	54706	1563951
diacyl			879883	3655	1318749
Alkyl-acyl			447960	51051	245202
% of Total					
diacyl			66%	7%	84%
Alkyl-acyl			34%	93%	16%

m/z, mass-to-charge ratio; RT, retention time

Values in the last three columns, intensity (counts per second (cps))

Table S2. Cells used in this study.

Cell line	Features	origin
HEK293	Wild type	ATCC CRL-1573
HEK293 ARV1KO	clone	This study
HEK293 PIGAKO		This lab (49)
HEK293 PIGQKO		
HEK293 PIGA/ARV1DKO		This study
HEK293 PIGA/ARV1-DKO-GST-PIGA-tetOn empty vector	Puromycin-, hygromycin-, and G418-resistance	
HEK293 PIGA/ARV1-DKO-GST-PIGA-tetOn FLAG-ARV1		
HEK293 PIGA^{R119W}KI	clone	This study
HEK293 PIGA^{R119W}KI/ARV1KO		
HEK293 PIGA^{R119W}KI/ARV1KO-tetOn empty vector	Puromycin- and G418-resistance	
HEK293 PIGA^{R119W}KI/ARV1KO-tetOn FLAG-ARV1	Puromycin- and G418-resistance	
HEK293 PIGO^{R119W}KI	clone	This study
HEK293 PIGO^{R119W}KI/ARV1KO		
HEK293 PIGT^{P183T}KI		
HEK293 PIGT^{P183T}KI/ARV1KO		This study
HEK293 PIGLKO		This lab (49)
HEK293 PIGL/ARV1DKO		This study
HEK293 PIGL/ARV1DKO-ARV1	Puromycin-resistance	
Human fibroblasts-Tert	Neomycin-resistance	This study
Human fibroblasts-Tert ARV1KO	Neomycin- and puromycin-resistance	
Human fibroblasts-Tert ARV1KO- ARV1^{Cas9r}	Neomycin-, puromycin-, and blasticidin-resistance	
Human fibroblasts-Tert ARV1KO- pLiB2 empty vector		
Expi293		Thermo Fisher
LentiX 293T		Clontech
PLAT-GP		(50)

Table S3. Oligonucleotides used in this study

Name	Sequence 5'-3'	Purpose
hARV1-KO1 mu F	TCGGCTTCCTGTCAGTACAGGTGCATCGAATG	Construct Cas9 protein resistant human ARV1
hARV1-KO1 mu R	CTGACAGGAAGCCGAGGCAGCAGTAGGAG	
hARV1-KO2 mu F	GTATAGAGACTACAACCACGGTGTGCTGAAG	
hARV1-KO2 mu R	TTGTAGTCTCTATACAACTCTTTGGCCTCCTGG	
pME-3HA-ARV1 F	GTCGAGGGCAACGGCGGG	InFusion primers
pME-3HA-ARV1 R	GGCGCCAGCGTAATCAGGC	
Sal1-ARV1 F	AAAAGTCGACATGGGCAACGGCGGGCGGAG	Amplify ARV1 to fit pME-6HisFLAG vector
Not1-ARV1 R	AAAAGCGGCCGCTCAGAAGTCCTGAGATTTAAAG	
ARV1-L53P F	CGGTGTGCCGAAGATAACCATCTGTAAATCCTGC	Construct ARV1 mutant expressing plasmids
ARV1-L53P R	ATCTTCGGCACACCGTGGTTATAGTCTCG	
ARV1-Δ53-137 F	ACGGTGTGTGGGATTTCTATAGAATGTTTGCG	
ARV1-Δ53-137 R	AATCCCACACACCGTGGTTATAGTCTCGG	
ARV1-K84A F	ATTGTGCGCAGCTCAGGCCTACAGACATATTC	
ARV1-K84A R	TGAGCTGCGCACAATATAGCATTAATCAAGATG	
ARV1-N79Q F	CTTGATTCAAGCTATATTGTGCAAAGCTCAGG	
ARV1-N79Q R	ATAGCTTGAATCAAGATGATAACAGGATCATAC	
ARV1-K84R F	ATTGTGCCGAGCTCAGGCCTACAGACATATTC	
ARV1-K84R R	TGAGCTCGGCACAATATAGCATTAATCAAGATG	
ARV1-Y71A R	AGGATCAGCCTCGATATATTTGTCTACAGGTTTC	
ARV1-Y71A F	ATCGAGGCTGATCCTGTTATCATCTTGATTAATGC	
ARV1-D72A F	ATCGAGTATGCTCCTGTTATCATCTTGATTAATGC	
ARV1-D72A R	AGGAGCATACTCGATATATTTGTCTACAGGTTTC	
ARV1-D72N F	ATCGAGTATAATCCTGTTATCATCTTGATTAATGC	
ARV1-D72N R	AGGATTATACTCGATATATTTGTCTACAGGTTTC	
ARV1-N79G F	CTTGATTGGTGCTATATTGTGCAAAGCTCAGG	
ARV1-N79G R	ATAGCACCAATCAAGATGATAACAGGATCATAC	
ARV1-KO1 F	CACCGATGCACCTGTACTGGCAGG	Cloning into pX330/ LentiCRISPR v2 for ARV1 knockout
ARV1-KO1 R	AAACCCTGCCAGTACAGGTGCATC	
ARV1-KO2 F	CACCGCACACCGTGGTTATAGTCT	
ARV1-KO2 R	AAACAGACTATAACCACGGTGTGC	
ARV1-KO Seq F	AGTTGAGTGGAAATGGGCAACGG	Genotyping for ARV1knockout clones
ARV1-KO Seq R	CGTTCGCCGGCTTAGGTCATAT	
